# A Novel Double Side Branch Protection Technique for a Left Main Trifurcation Lesion: Simultaneous Jailed Balloon and Jailed Corsair Technique

**DOI:** 10.1155/2018/6852946

**Published:** 2018-09-13

**Authors:** Masahito Munakata, Yohei Numasawa, Shiro Ishikawa, Takashi Koyama

**Affiliations:** ^1^Department of Cardiology, Saitama Municipal Hospital, Saitama, Japan; ^2^Department of Cardiology, Japanese Red Cross Ashikaga Hospital, Ashikaga, Japan

## Abstract

Percutaneous coronary intervention for left main trifurcation disease is challenging. Although side branch protection techniques such as the jailed balloon technique and jailed Corsair technique are the established methods for treatment of coronary bifurcation lesions, little is known regarding the application and feasibility of these techniques for left main trifurcation disease. We herein describe a 72-year-old man with angina pectoris who was successfully treated with percutaneous coronary stent implantation for a left main trifurcation lesion. In this case, we performed a novel double side branch protection technique, the simultaneous jailed balloon and jailed Corsair technique, with a single 8 Fr guiding catheter. This is the first case report to highlight the feasibility and efficacy of combined use of the jailed balloon and jailed Corsair techniques during percutaneous coronary intervention for left main trifurcation disease.

## 1. Introduction

Percutaneous coronary intervention (PCI) for left main trifurcation disease remains one of the most technically challenging procedures for interventional cardiologists, even with the currently available drug-eluting stents (DESs). In general, provisional stenting using the jailed wire technique is considered the standard method for most coronary bifurcation lesions [1]. However, even when a protective guide wire is inserted into the side branch prior to main vessel stenting, there remains a risk of side branch occlusion after stent implantation due to plaque or carina shift [2, 3]. To overcome these difficulties associated with PCI for bifurcation lesions, the jailed balloon technique and jailed Corsair technique have been established [3–9]. Although these techniques are useful, PCI is more complex for coronary trifurcation than bifurcation lesions [10–13], and little is known regarding the application and feasibility of these side branch protection techniques for left main trifurcation disease. We herein describe a 72-year-old man with angina pectoris who was successfully treated with percutaneous coronary stent implantation for a left main trifurcation lesion using the simultaneous jailed balloon and jailed Corsair technique.

## 2. Case Presentation

A 72-year-old man presented to our hospital with a chief complaint of chest pain on exertion. He had multiple coronary risk factors including hypertension, dyslipidemia, and diabetes mellitus. Electrocardiography revealed no clear findings of ischemic ST-T changes or prior Q-wave myocardial infarction. Transthoracic echocardiography revealed an ejection fraction of 63.3% with mild left ventricular hypertrophy and no segmental wall motion abnormality. Coronary computed tomography angiography revealed a mixed plaque in the distal left main trifurcation with suspicion of significant stenosis. He was admitted to our hospital to undergo coronary angiography with a diagnosis of stable exertional angina pectoris.

**Figure 1:**
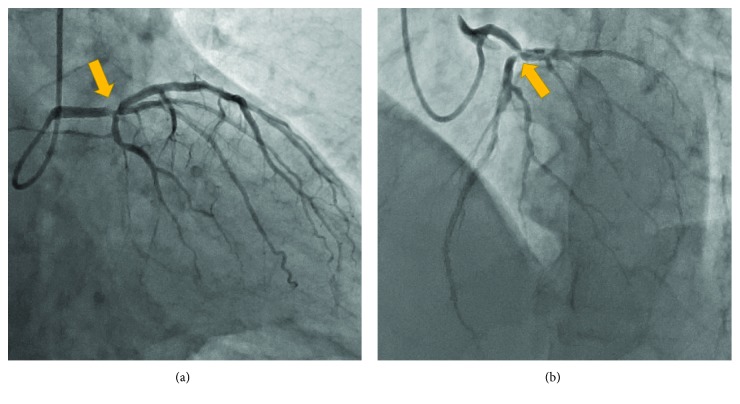
Left coronary angiogram of the (a) right anterior oblique caudal view and (b) left anterior oblique cranial view revealed a significant distal left main trifurcation lesion (yellow arrows).

Diagnostic coronary angiography revealed distal left main trifurcation disease including significant stenosis in the distal left main trunk (LMT), ostial left anterior descending artery (LAD), and ostial left circumflex artery (LCX) (modified Medina classification 1-1-1-0) (Figure 1) [11]. No significant stenosis was present in the right coronary artery. Because the SYNTAX score was calculated as 15 [14] and the patient refused a surgical approach, PCI using a DES was planned. Because both the LCX and intermediate branch were large vessels, it was essential to preserve the blood flow of these side branches after main vessel stenting. Therefore, we decided to perform PCI using the simultaneous jailed balloon and jailed Corsair technique for this left main trifurcation lesion.

**Figure 2:**
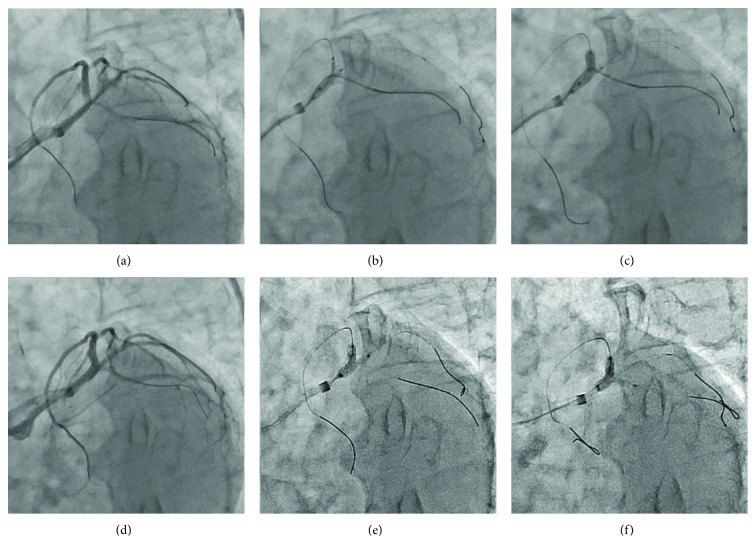
Stent implantation using the simultaneous jailed balloon and jailed Corsair technique. (a) Left coronary angiogram of the left anterior oblique caudal view before stent implantation. (b) The side branch balloon in the intermediate branch was initially inflated. (c) The main branch stent balloon was inflated, which jailed the side branch semi-inflated balloon in the intermediate branch and the Corsair Pro microcatheter in the left circumflex artery. (d) Blood flow was preserved in both side branches after stenting. (e, f) Final kissing balloon inflations were performed.

After obtaining informed consent, PCI for the left main trifurcation lesion was performed. An 8 Fr AL 1.0 guiding catheter with a side hole (Hyperion; Asahi Intecc, Aichi, Japan) was engaged into the left coronary artery via the right femoral artery. A SION guide wire (Asahi Intecc) was initially inserted into the LAD. To protect the two large side branches, a Balance Middleweight Universal II guide wire (Abbott Vascular Japan, Tokyo, Japan) and a SION Blue guide wire (Asahi Intecc) with a Corsair Pro microcatheter (Asahi Intecc) were introduced into the intermediate branch and LCX, respectively (Figure 2(a)).

According to the angiographic and intravascular ultrasound images, we decided to perform direct crossover stenting from the LMT to LAD using the simultaneous jailed balloon and jailed Corsair technique. A DES (SYNERGY 3.0 × 16 mm; Boston Scientific, Natick, MA, USA) was advanced into the LAD, and a semicompliant balloon (canPass 2.5 × 15 mm; Japan Lifeline, Tokyo, Japan) was advanced into the intermediate branch. Subsequently, a Corsair Pro microcatheter was introduced into the LCX. The simultaneous jailed balloon and jailed Corsair technique was then performed. The side branch balloon in the intermediate branch was initially inflated at low pressure (3 atm) (Figure 2(b)). The main branch stent balloon in the LMT to LAD was then inflated three times at nominal pressure (11 atm), which simultaneously jailed the side branch semi-inflated balloon in the intermediate branch and the Corsair Pro microcatheter in the LCX (Figure 2(c)). When the jailed Corsair Pro microcatheter was removed with rotation of the shaft, only small friction was felt. Blood flow was preserved in both the intermediate branch and the LCX after main vessel stenting, and there were no signs of plaque or carina shift into the side branches (Figure 2(d)).

**Figure 3:**
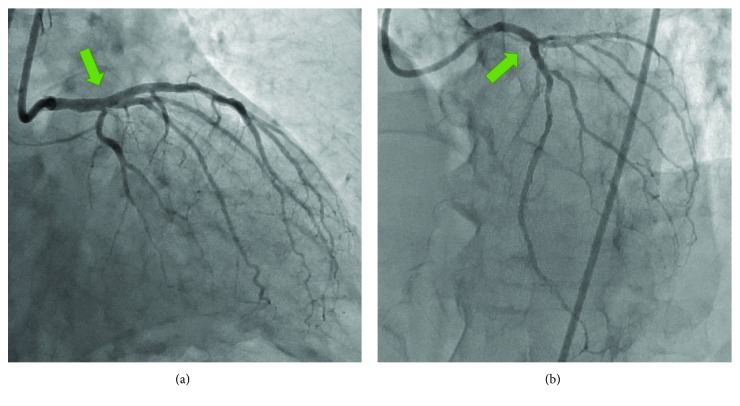
Final coronary angiogram of the (a) right anterior oblique caudal view and (b) left anterior oblique cranial view showed excellent results without any complications including side branch narrowing or occlusion (green arrows).

After reinsertion of a SION Black guide wire (Asahi Intecc) into the jailed intermediate branch via stent struts using a double-lumen catheter (Crusade K; Kaneka Medix, Osaka, Japan), kissing balloon inflation was performed with a 3.0 × 16 mm stent balloon in the LAD and a 2.5 × 15 mm canPass balloon in the intermediate branch (Figure 2(e)). Final kissing balloon angioplasty was then performed with a 3.0 × 16 mm stent balloon in the LAD and a 2.5 × 15 mm canPass balloon in the LCX (Figure 2(f)). A final coronary angiogram showed excellent results without any complications, including side branch narrowing or occlusion (Figure 3). The postprocedure course was uneventful, and no major complications including ischemic events, heart failure, or access-site complications were observed. The patient was discharged the day after the procedure, and his angina symptom completely disappeared after PCI.

Because left main trifurcation was anatomically crucial, an early follow-up coronary angiography was performed 2 weeks after the procedure. It showed excellent results and no findings of restenosis in both the main vessel and side branches. In addition, fractional flow reserve (FFR) was measured and there were no signs of significant ischemia in both distal LAD (FFR: 0.88) and LCX (FFR: 1.00).

## 3. Discussion

This case report highlights the feasibility and efficacy of the simultaneous jailed balloon and jailed Corsair technique with a single 8 Fr guiding catheter for the treatment of left main trifurcation disease. To the best of our knowledge, this is the first case report detailing successful percutaneous coronary stent implantation using this technique.

PCI for coronary bifurcation or trifurcation lesions, especially with left main trunk disease, remains challenging and is associated with a risk of side branch occlusion and a high rate of target lesion revascularization [11, 12]. The provisional stenting method using the jailed wire technique is generally recommended for most coronary bifurcation lesions [1]. However, side branch occlusion or side branch narrowing after main vessel stenting sometimes occurs because of plaque or carina shift, even when a protective guide wire is inserted into the side branch prior to stent implantation [2, 3]. Once the blood flow of the side branch is impaired, serious myocardial ischemia may develop along with chest pain and hemodynamic instability, even when the blood flow impairment is temporary [13]. Therefore, efforts to reduce the risk of temporary or chronic side branch occlusion during PCI are crucial. In addition, PCI is more complex for coronary trifurcation than for bifurcation lesions [10–13]. Although coronary artery bypass grafting surgery is generally recommended for patients with left main trifurcation disease, especially those with ostial plaque involvement of side branches, PCI may sometimes be required in selected patients with acute coronary syndrome at presentation and intolerance of or refusal to undergo a surgical approach mainly because of old age or systemic comorbidities [13].

To overcome difficulties associated with PCI for bifurcation lesions, the jailed balloon technique and jailed Corsair technique have been established [3–9]. The jailed semi-inflated balloon technique provides a high rate of procedural success and preservation of the side branch blood flow after main vessel stenting mainly because it prevents carina or plaque shift [5, 8]. However, this technique is associated with potential risks including jailed balloon entrapment, balloon rupture, and dissection of the side branch ostium by balloon inflation even with low pressure. Compared with the jailed balloon technique, the jailed Corsair technique is more suitable for protecting a small side branch, and the risk of dissection at the ostium of the side branch is lower [9]. In addition, because the shaft of the Corsair Pro microcatheter has both high strength and excellent lubricity due to its hydrophilic coating, the risk of device entrapment is minimal. The concept of the jailed Corsair technique is consistent with the “keep it open strategy” stated in the consensus document of the European Bifurcation Club [1]. Taking these advantages and disadvantages of the jailed balloon technique and jailed Corsair technique into account, the combined use of these two techniques for left main trifurcation disease may be reasonable. Because the intermediate branch was larger than the LCX in this case, we simultaneously performed the jailed balloon technique in the intermediate branch and the jailed Corsair technique in the LCX during main vessel stenting.

Our case report indicates that the basic treatment concepts for most coronary bifurcation lesions can be applied to trifurcation lesions including the jailed balloon and jailed Corsair technique. A provisional strategy with a single stent may also be a standard method, even in patients with left main trifurcation disease [11, 12], although the two side branches should be protected separately. In this situation, the simultaneous jailed balloon and jailed Corsair technique with a single 8 Fr guiding catheter is a useful alternative, as shown in this case report.

The simultaneous jailed balloon and jailed Corsair technique has some limitations. First, this technique requires an 8 Fr guiding catheter for simultaneous insertion of a stent delivery system, balloon catheter, and Corsair microcatheter. Therefore, this technique cannot be performed via the radial artery. Second, because the proximal marker of the side branch balloon should be located more proximal to the stent edge to prevent entrapment [6, 8], a long balloon with double radiopaque markers is necessary. Finally, the use of a semi-inflated balloon or Corsair microcatheter for side branch protection is off-label, and it may also be associated with an increase in the total procedural cost.

## 4. Conclusion

The simultaneous jailed balloon and jailed Corsair technique is a novel and effective double side branch protection technique for the treatment of left main trifurcation disease in selected patients. Further studies on this technique in larger populations are needed.
